# Mainstreaming health and wellness: The RHWP Innovation model to complement primary care

**DOI:** 10.1111/nuf.12326

**Published:** 2019-01-29

**Authors:** Pamela L. Parsons, Patricia W. Slattum, Michael Bleich

**Affiliations:** ^1^ Department of Family and Community Health Nursing, Virginia Commonwealth University Richmond Virginia

## Abstract

Problem statement: Multiple chronic conditions combined with the complex social needs of individuals and families often create unattainable goals of efficient and effective holistic care within primary care settings. There is a recognized need for new approaches to address the intersection of the role of social determinants of health and the resulting impact on health care utilization and outcomes as an approach to enhancing value‐based care. Model description: This paper describes an innovative health and wellness model that complements the essential work of primary care providers (PCPs), as an adjunct to care delivery. The wellness program helps meet unrealistic expectations placed on providers to cover a full range of holistic services while reducing the burden on under‐ or uninsured patients to seek timely care. The model describes an academic‐community based partnership that integrates student learning into the delivery of a wellness program provided on‐site to adults residing in apartment buildings designated for low‐income and disabled adults. The innovation described is a health and wellness model that complements the demands placed on primary care clinics.

Primary care is the foundation that underlies clinical care in the U.S. health system. Managed by physicians and advanced practice registered nurses, other professions such as pharmacists now play roles in becoming access points for people needing essential clinical care. Primary care providers (PCPs) in clinic and ambulatory settings perform broad‐based care independent of specialists, care covering a spectrum of conditions ranging from infectious diseases to high‐risk diagnosis and management of acute and chronic conditions, lifespan health prevention and adjustment concerns, and much more. The demands of individuals and families seeking primary care create clinic settings that are rapid‐paced, chaotic, and sometimes unpredictable; these issues are exacerbated in regions where provider shortages exist. The coordination activities required to complete referrals to specialists and referral agencies add even more time to PCP responsibilities. The literature describes the PCP as a gateway to assimilating comprehensive care; however, a history of poor outcomes at the individual and health system level has led to the development of innovative models of integrating value‐based holistic care into health care reform.^1^

This paper presents an innovative health and wellness model that complements the essential work of PCPs. The model is an adjunct to primary care delivery that makes clinical and fiscal sense, helps meet unrealized expectations placed on providers to cover the range of services patients expect, reduces the burden on under‐ or uninsured patients to seek timely care that may be unaffordable, and improves linkages to address social determinants of health that impact health outcomes. Health is defined in this model as one's physical, mental, and social peace of mind, an absence of distress, and, when the disease has been diagnosed, is treated and not advancing to a higher level of complexity (WHO, University of Buffalo).^2^ Wellness is defined as a state of optimal well‐being in physical, intellectual, interpersonal, spiritual, social, occupational, and emotional dimensions. Wellness promotes freedom of expression with others, affording an ability to cope, take responsibility, live peacefully, and continue development as life's demands unfold (National Wellness Institute).^3^

## 1 BACKGROUND AND SIGNIFICANCE

Nationally, nearly 800,000 older adults reside in low‐income housing, receive federal rental assistance, and are challenged by environmental factors and social determinants of health (The Pew Charitable Trusts 2015).^4^ About half of these individuals are disabled, functionally impaired, and have high chronic disease burden; 66% are obese and 25% are diabetic.^5^, ^6^ Unmanaged health conditions, coupled with social barriers to accessing health care, can result in increased nonurgent emergency room use, hospitalization, or admission to higher cost assisted living or nursing homes. As noted, PCPs have competing demands placed on them when diagnosing and managing patient disease conditions and are even more challenged when they account for their patient's complex environmental and social issues. We believe our wellness innovation model offloads the nondiagnostic demands on PCPs and leads to population health practices that are efficient and effective.

Within the Richmond region, seven percent of individuals live below the poverty level and reside in neighborhoods with limited access to transportation, have high crime rates, and have finite access to basic shopping and nutritional food. Dong et al, Parsons & Boling, Wise et al,^7^, ^8^, ^9^ with roots in primary and community‐based care, faculty providers from the Virginia Commonwealth University, health professional schools of nursing, pharmacy, medicine, social work, allied health, and psychology developed a strategy to foster aging in place, complement primary care, and reduce the human and resource utilization toll on individuals and the health system. The Richmond Health and Wellness Program (RHWP) was launched in 2012.

## 2 PROGRAM MODEL: THE (CITY NAME) HEALTH AND WELLNESS PROGRAM

The RHWP aligned with national efforts to address the social determinants of health that impede daily living, factors known to be associated with over 70% of poor outcomes in our city of Richmond Anti‐Poverty Report, 2013.^10^ Recognizing healthcare as a complex adaptive system that interconnects and is multi‐layered, the provider faculty met with 249 old and disabled residents in an urban, low‐income apartment complex in Richmond (County Health Rankings 2018)^11^ to determine the value and interest by residents of a place‐based intervention. Using the complexity strategy of cocreation, relationships developed through persistent conversations and onsite presence, generating mutual trust between residents and providers from which the program model emerged.^12^ Health promotion and wellness services coupled with resource coordination among primary care and other community service agencies emerged as priorities; residents supported a nursing care model with an interprofessional health team (IPT) to best serve their needs. A nursing model suited the goals at hand because it (a) supported holistic care; (b) accommodated health promotion and disease prevention; (c) was adaptable and unified all health disciplines; and (d) supported evidence‐based practice with a focus on individuals, families, and communities. In 2013, the RHWP received HRSA Nursing Education, Practice, Quality and Retention Program funding, furthering program expansion to four additional low‐income apartment buildings, with service capacity to over 500 residents. Today the program operates in five facilities one half‐day to a full‐day weekly based on resident volume in each housing facility. Nearly 60% of the RHWP enrollees are enrolled in Medicare or Medicaid. The enrollees are primarily female (58%) and African American (72%). Sixty‐one percent are over 65 years of age, 33% did not complete high school, and 42% have a monthly income of less than $1,000. Over 90% had a PCP at the time of enrollment into the program.

**Figure 1:**
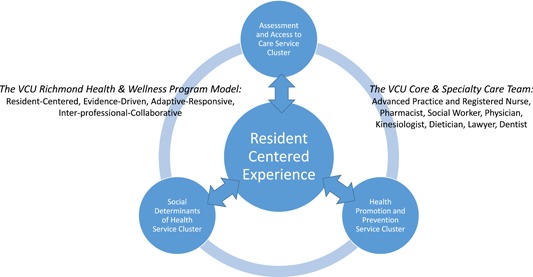
The Virginia Commonwealth University Richmond Health & Wellness Program model [Color figure can be viewed at wileyonlinelibrary.com]

From the onset, residents gave voice to the program's design and content, which led to what are now quarterly resident council meetings to share and tailor program changes and evaluate impact. Initially, providers offered expertise to the residents who expressed their preferences in town hall meetings, a precursor to the resident council. The first priorities residents set centered on getting help with diabetes and blood pressure monitoring, managing prescribed medications, pursuing transportation to medical care, and, more broadly, exploring ways to assure their mental health and general wellbeing. Providers yielded to these resident‐centered needs, the program was launched, and plans were established to grow the program with health professions students performing service learning and seeking community agency partnerships to lend technical expertise, share services without duplication, and offer financial support. The model in Figure 1 depicts the current three clusters of service offerings, philosophical principles used in the approach to care, and the interprofessional providers have expanded to constitute the current care team. Each is described below.

### 2.1 Resident‐centered care

The catalyst for a resident‐centered, community engagement approach is centered in the patient‐centered care movement. In this approach, active collaboration and shared decision‐making was believed to increase satisfaction among the residents and providers alike, improve resource allocation, drive care coordination by respecting residents’ values, preferences and needs, and increase information flow, communication and education (NEJM Catalyst, 2017).^13^ Sillars^14^ goes on to state that resident‐centered care is team based, views the resident in a holistic manner through a human rather than disease lens, drives healing through the relationships established, and removes stigma and judgment, with a problem and goal focus that is in the functional language of the resident. While programs focused on population needs, service delivery addressed individuals honored by the interprofessional team.

### 2.2 Assessment and access to care

Residents may freely choose to use any or all the services offered and can enroll in the RHWP at any time. The sole requirement is that on enrollment a comprehensive geriatric baseline assessment is completed to ascertain the resident's medical and social history, medication profile, cognition and functional status, and vulnerability status for depression, food insecurity, and frailty. Further, care navigation needs are assessed to improve access to care and medical communication. Assessments involve the use of a variety of valid and reliable instruments to determine the resident's health status, risk factors, and medical and social history. Interprofessional students conduct the assessment after training and competency validation led by faculty experts with primary care, community, and geriatric specializations. The assessment is the basis for personalized care planning, measuring status changes, and priority‐setting.

### 2.3 Health promotion and prevention service cluster

Residents acknowledge that remaining healthy directly impacts the quality of their lives. Given their status as older adults, many residents had a desire achieve higher levels of self‐care management to bridge existing chronic conditions, prevent disease progression, and complications/side effects associated with medical treatment, such as falls, and improve overall social wellbeing. After hospitalization or emergency treatment, residents had heightened concern over readmissions.

Program participants select interventions from a menu of options delivered individually or in groups by interprofessional program team (IPT) members. Medication review, health and safe living assessment and monitoring, individual counseling, and coaching to optimize health status reporting to their PCPs all emphasize health promotion and disease prevention. A faculty member with a longitudinal resident relationship matches IPT resources to the resident's needs. For instance, for residents struggling with medication‐related issues, be that in procuring and paying for drug therapy, achieving therapeutic intent, living with side effects, or managing a complex drug regimen may have a pharmacy student and an advanced practice nursing student assigned by faculty to mitigate resident concerns as a team. Or, a nursing and social work student might conduct a home visit to assist someone functionally impaired, having a decline in health, to conduct a safe living analysis, or follow a return from the hospital, emergency room, or nursing home.

### 2.4 Social determinants of health‐related services

Social determinants of health are the conditions in which people are born, grow, live, work, and age. They include factors that influence economic stability, the neighborhood and physical environment, access to education and food, communal and social resources, and availability of health care resources. Service‐related programs that confronted negative social determinants in these neighborhoods include offering congregate hot meals (offsetting the absence of grocery stores), legal support to provide assistance with establishing advanced directives and wills, encouraging social activities to reduce social isolation, and create a sense of community, providing financial planning given the impact of poverty, and promoting stable housing, a major destabilizing problem for this population. As noted by Desmond,^15^ the home is the center of life, where safety and security are fostered, where civic life begins, and where psychological stability is formed. Disruptions in the home cause disruptions in healthy living and wellness and can impede access to primary care. For these reasons, the model was first applied in housing facilities.

### 2.5 Impact and lessons learned

The RHWP has impacted the quality of life for the residents it has served. It has also been touted by PCPs for being nonduplicative of their efforts and improving resident‐to‐provider communication, filling in service gaps, and tending to the long‐term consequences of vulnerable older and disabled adults.

### 2.6 The importance of care coordination and home visits

The care coordination and intermittent home visit services are two of the programs high impact contributions. Especially, the IPT who coordinates care and conducts home visits provides residents with

a. Reliable clinical data for residents to share with their PCPs, such as blood pressure, glucose testing results, and other health sourced data that is trended in the clinics.
b. Coaching on how to communicate medical information with their provider so that clinical problems are adequately addressed. This information can range from the side effects of drug therapy to disease‐specific information. Residents learn how to ask for clarification in their medical treatment.
c. Resources to get to and from appointments, decreasing missed visits.
d. Ongoing observations in weekly clinics that detect changing clinical conditions, thereby advising residents to get care before they are in a clinical crisis. If an acute issue is detected, immediate contact with the PCP is activated.
e. Support for achieving “big picture” interagency resource coordination and alignment, between primary care and community agencies that can contribute to the resident's quality of life with a more singular, yet valuable, purpose.
f. Posthospitalization checks in the resident's apartment clinically reinforce discharge planning, assist with medical follow‐up (including a review and acquisition of medications), and promote safety.

Other services are organized through weekly clinics held at each housing complex in donated community space. Using space dividers, four teams offer concurrent wellness consultations on a first‐come, first served basis. Nursing student is trained to triage the residents, followed by a care coordinator assigning each resident, based on their identified needs, to a team most suited as a match to the resident's circumstance. If the need is an illness rather than wellness oriented, a nurse practitioner student then assesses the resident's status with an eye toward early PCP intervention. As the visit concludes, the IPT determines the root causes of new issues and establishes goals requiring follow‐up and, if necessary, care coordination interventions are placed in motion. Residents are reminded of communal programs and events that are forthcoming. Licensed clinical faculty partners with students throughout the process, taking responsibility for postvisit communications with PCP and/or partnering community stakeholders.

### 2.7 The Importance of the Interprofessional Care Team

**Figure 2:**
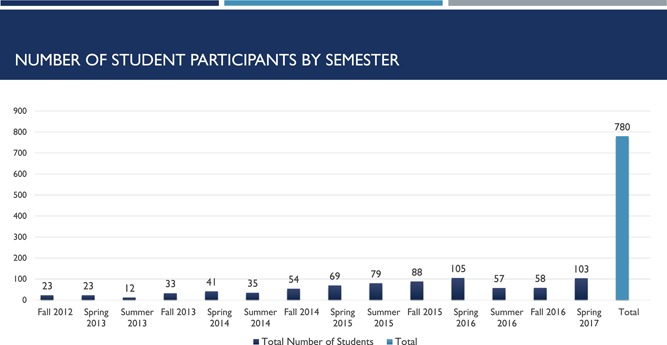
Number of Student Participants by Semester [Color figure can be viewed at wileyonlinelibrary.com]

**Figure 3:**
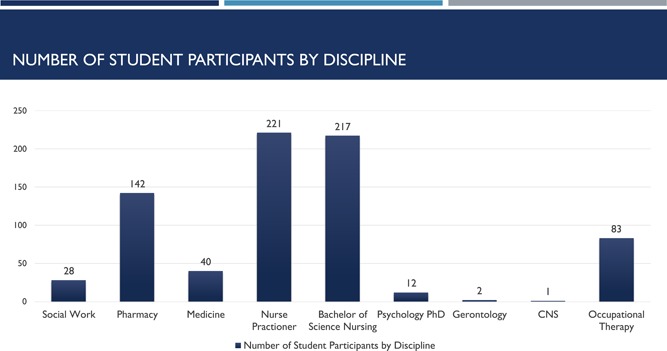
Number of student participants by discipline [Color figure can be viewed at wileyonlinelibrary.com]

Strong student engagement is reflected with the engagement of 780 interprofessional team members through the Spring of 2017 (see Figure 2) with the majority comprised of nursing, followed by the pharmacy (see Figure 3). Experienced delivering community‐based care to lower‐income or disabled adults residing in one of the five housing complexes. The original care team, comprised of advanced practice and registered nurse students along with a pharmacy, social work, and medical student colleagues each represent disciplines congruent with the program definitions of health and wellness described earlier and in concordance with resident desires for services. As the program matured and expanded, dieticians and kinesiologists from the School of Allied Health joined the team. A private law school from outside our university added another new dimension to resident services in the past year and in the future dentistry will play a more prominent role in community‐based care.

All students within the (university) health system receive six university credits (two courses) of training in interprofessional team‐based care. An outcome of the RWHP is that it provides students with applied learning and enriched geriatric content through structured team conferences. Motivational interviewing, holistic assessment, TEAMSTEPPS, and other content ensures interprofessional competencies in population health and team‐based care delivery. Further, students provide resident education that is age, cultural, and literacy‐level appropriate in wellness‐based subjects such as nutrition and healthy eating, weight loss, medication adherence, exercise, and smoking cessation.

IPT students are also immersed in the challenges that people face in managing their overall wellbeing in a setting outside the healthcare settings where their training has taken place. For instance, during home visits, the problem of food insecurity becomes strikingly real, as residents were forced to choose between affording food or their medications. The significance of meals on wheels and the weekly communal meal was elevated, not trivialized. Social isolation from lack of transportation, neighborhood violence, and other family‐based factors led to keen insights about the role and functions of families, spirituality, and safety. Functional impairments not as readily noticeable in acute care settings were now seen as a path to loss of independence and housing.

Residents appreciate the intergenerational presence of students. The clinics and programs offered by RHWP provide cognitive and social stimulation to their lives. Relationships are established not only with students but with faculty who manage a caseload of residents longitudinally. Trust and rapport between the residents and RHWP providers are readily evidenced and cited as a program strength.

### 2.8 The importance of community partnerships

The RHWP had been undoubtedly enriched by community‐based partnerships. The tendency to replicate services between agencies adds chaos to the choices residents must make, despite well‐meaning intentions. In this model, such duplication is minimized. Rather, a concerted study of agency intentions that align with resident preferences has led to trusted dependencies on local food banks, the local area agency on aging, homeless collaboratives, and a private law school with shared mission for service and training. Rather than being perceived as competitors, these partnerships are valued as pathways to feed, transport, secure stable housing, restore credit, renew driver's licenses, and grant power of attorney to residents. They are combatant partners in shaping policy, securing grants, and offsetting negative social determinants of health, all positive outcomes. The socialization and cocreation of the program *with* residents, with students, with providers, with community agencies, and with institutional leaders have created a synergistic learning environment where the power of connections is realized.

### 2.9 The importance of sustainability

The RHWP has been sustained through the University and each school that has funded faculty practice. In return, each school has a received the benefit of training opportunities for its students, grant award, and community presence, consistent with the University's mission and goals.

Health and wellness programs are not heavily dependent on high‐cost technology and massive space demands. Each housing facility has its own administrator and may vary in its regulatory structures, so it takes the effort to develop administrative partners who are willing to offset rental space and free their staff to support whatever coordination requirements are necessary. In turn, each housing facility has access to an array of professional resources they might not otherwise have to support resident life.

Sustainability, we learned, requires program evaluation data. Early data sets included enrollment data, service encounters, and formative evaluations with residents. Beyond the scope of this paper, the RHWP has evolved its evaluation to include new statistical methods that can prove the economic impact of the program by calculating actual with predicted service risk‐adjusted utilization patterns using population‐level data. Our pilot study demonstrates fewer hospitalizations and emergency department visits, suggesting that our prevention‐oriented services offset crisis clinical management. This pilot study provided an entrée to discussions that would lead to a sustainable per‐member‐per‐month contract with local insurers, building the business case for funding programs that are prevention and quality‐of‐life based.

## 3 SUMMARY

This paper has presented a health and wellness model that is serving the needs of residents in five housing facilities serving low income and disabled older adults. We have presented the key principles that allowed the model to develop and the use of an interprofessional health care team. We have supported the use of health professions students as agents for service learning, sensitizing these students to a community‐based practice that is far removed from the acute care environment where most training occurs.

Beyond the impact on the residents in each facility and to the student experience, our partnerships have led us to external tables where we have been able to engage in payment reform discussions to fund community‐based care, ways to more efficiently and effectively organize community services, integrate older adults into the broader community, and increase healthcare and social service workforce training in population health.

The model is innovative in the scope of services provided, the scalable use of housing facilities to meet IPT training needs in population health and individualized care, the profound awareness of the impact of social determinants of health in pursuing desired conditions to pursue health and well‐being, and in creating program evaluation models that extend into cost‐effective care. In the near future, the model will guide the establishment of a health and wellness center that is not space‐based, but neighborhood based. This new center commits to the principles defined in the RHWP model and will eliminate a food desert by adding grocery and other services that have robbed individuals from neighborhood features that are life uplifting. As an adjunct to primary care, we have demonstrated that holistic approaches at the individual/family level that are community‐based can improve the primary care experience for both providers and patients.

This manuscript is dedicated to the memory of a nurse leader who believed strongly in the humanity of nursing, interprofessional care, and elevating people to their highest form of functioning, Jeffrey Petraco.
